# A case of pituitary apoplexy in pregnancy

**DOI:** 10.1530/EDM-14-0043

**Published:** 2014-06-01

**Authors:** Aimee R Hayes, Anthony J O'Sullivan, Mark A Davies

**Affiliations:** 1 Departments of Endocrinology and NeurosurgerySt George Hospital and University of New South Wales, KogarahSydney, New South Wales, 2217Australia

## Abstract

Pituitary apoplexy is a rare event in pregnancy. A 41-year-old woman with a known pituitary microadenoma presented with visual disturbance and headache during the second trimester of pregnancy. Magnetic resonance imaging (MRI) demonstrated pituitary apoplexy with chiasmal compression. After treatment with corticosteroid therapy, she underwent transsphenoidal excision of the pituitary adenoma. Visual abnormalities were completely restored and pituitary function preserved. There was no evidence of impact on the foetus. The literature on the subject is reviewed with emphasis on the management of the apoplectic patient with mild and stable neuro-ophthalmological signs.

**Learning points:**

- There are no clear guidelines on the management of pituitary apoplexy in pregnancy. A multidisciplinary approach can minimise morbidity and mortality.
- Pituitary apoplexy has an unpredictable clinical course and determining which clinical situations warrant early surgery needs to take into consideration the presence and severity of neurological signs and their stability.
- The management of conscious apoplectic patients with absent or mild and stable neuro-ophthalmological signs is controversial.

## Background

Pituitary apoplexy is a potentially life-threatening event if not recognised early, although in recent decades mortality is unusual. It results from haemorrhagic infarction of a pre-existing pituitary adenoma or within a physiologically enlarged gland (1) (2). Rapid growth of pituitary tumours in pregnancy is well documented, although pituitary apoplexy is a rare event in pregnancy. Our case report describes a woman who presented with pituitary apoplexy in the second trimester of pregnancy.

## Case presentation

A 41-year-old woman, with a known pituitary microadenoma, presented with headache and visual disturbance at 18 weeks of gestation. She initially saw her chiropractor. It was only after seeing an ophthalmologist she became convinced that it was more than her neck causing the problem and an urgent endocrine and neurosurgical review was arranged.

**Figure 1:**
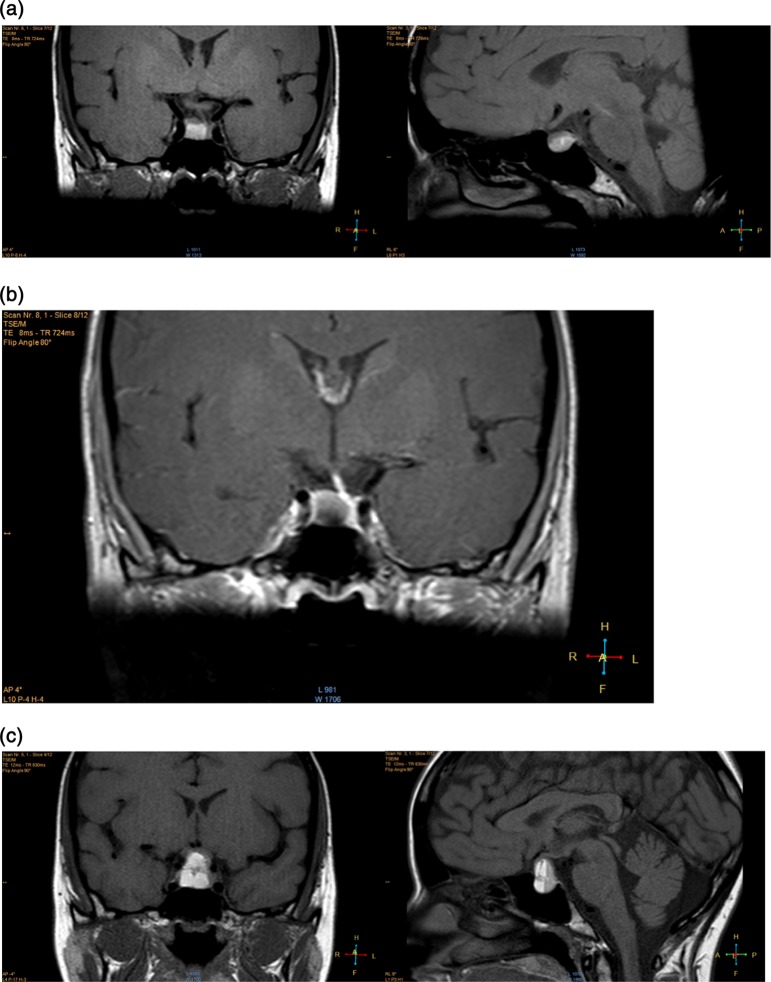
(a) Sagittal and coronal T1-weighted MRI scans showing cystic lesion in the central and right side of pituitary, consistent with microadenoma (6 mm in height and 9–10 mm transversely). The infundibulum is deviated to the left. (b) Gadolinium-enhanced T1-weighted coronal pituitary MRI; (c) sagittal and coronal T1-weighted MRI showing pituitary haemorrhage with a significant interval increase in the size of the adenoma (16–17 mm in height) and optic chiasmal compression.

Investigations for infertility and an irregular menstrual cycle performed two years previously at another facility, revealed a moderately elevated prolactin level varying from 1300 to 3000 mIU/l (40–570 mIU/l), raising the possibility of a prolactinoma, non-secretory adenoma with stalk compression or drug-related causes. She was taking no medications and was otherwise well with no comorbidities. Magnetic resonance imaging (MRI) demonstrated a cystic pituitary microadenoma with stalk deviation (Fig. 1a and b). Her vision was normal. Although she was thought to have a non-functioning microadenoma, she was advised to commence a trial of cabergoline and delay pregnancy. She did not commence cabergoline and did not pursue follow-up at this time.

At seven months before her presentation with apoplexy, she saw another endocrinologist and a neurosurgeon who both recommended a trial of cabergoline and to delay pregnancy. Pituitary MRI demonstrated a stable appearance. She commenced cabergoline 500 μg weekly but was unfortunately again lost to follow-up.

She subsequently became pregnant and was referred to our facility by her ophthalmologist. She had ceased the cabergoline once she realised that she was pregnant.

On presentation, her blood pressure was 120/74 mmHg with no evidence of a postural drop. There were subtle bitemporal visual field deficits to confrontation but no ophthalmoplegia or diplopia was reported. Remainder of her neurological examination including fundoscopy was normal. Systemic examination was unremarkable and consistent with an uncomplicated pregnancy of 18 weeks of gestation.

## Investigation

Non-contrast MRI demonstrated pituitary haemorrhage with a significant interval increase in the size of the adenoma (16–17 mm in height, compared with 6 mm before 7 months (Fig. 1c)). There were significant suprasellar extension and compression of the optic apparatus. Automated perimetry confirmed a bitemporal defect in the superior visual field. Haematology, biochemistry and coagulation profiles were normal. Pituitary hormone profile revealed thyroid stimulating hormone 1.5 mU/l (0.1–3.8 mU/l), free thyroxine 12.4 pmol/l (10.5–17.7 pmol/l), cortisol 875 nmol/l (155–599 nmol/l), insulin like growth factor-1 11 nmol/l (13–47 nmol/l) and prolactin 1539 mU/l (0–536 mU/l).

## Treatment

Urgent neurosurgical, endocrinology and obstetric team consultations were performed. Transsphenoidal surgery was felt most appropriate given the acute visual decline and unclear history whether the adenoma was sensitive to dopamine agonist therapy. The obstetric team advised that while there was a small risk of miscarriage with general anaesthesia, in the second trimester it was unlikely to be above background miscarriage rates. The risk of miscarriage associated specifically with transsphenoidal surgery was unknown.

**Figure 2:**
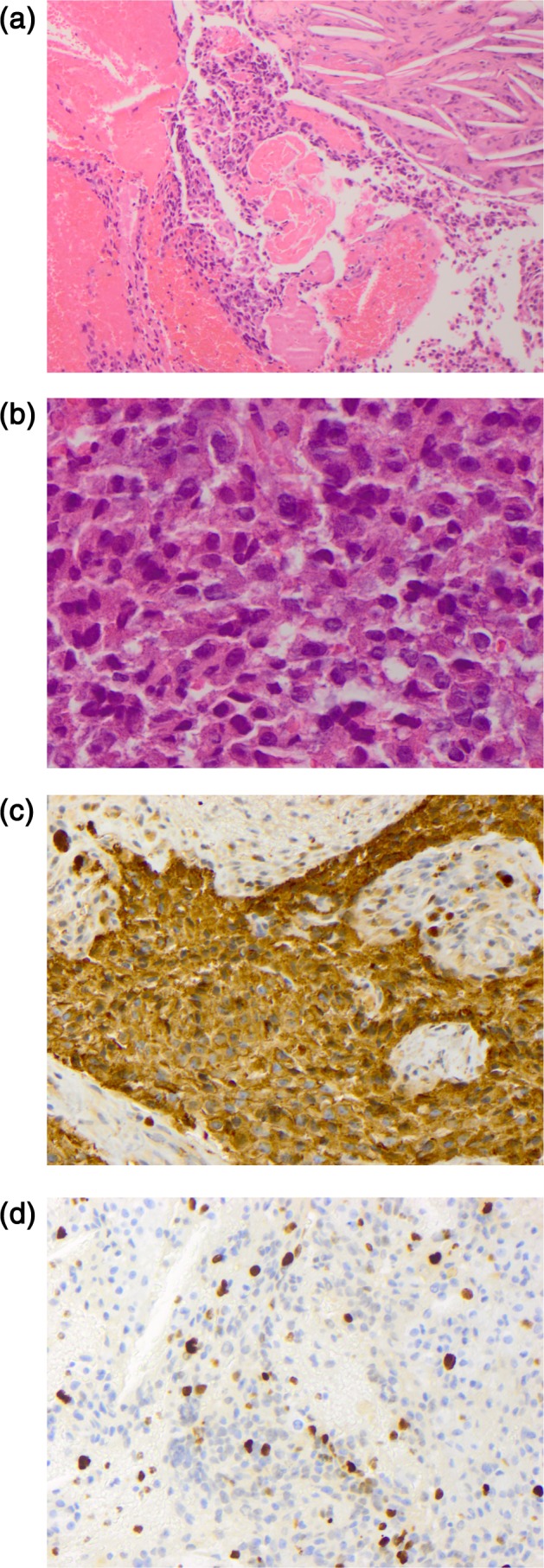
Histopathology from transsphenoidal resection of pituitary adenoma complicated by apoplexy. (a) Low power: prolactinoma with evidence of recent and prior haemorrhage (H&E). (b) High power: prolactinoma detail. Note granular eosinophilic cytoplasm (H&E). (c) The tumour shows ubiquitous expression of the prolactin immunohistochemical marker. (d) MIB1/Ki-67 proliferation index was low (5%).

High-dose corticosteroids were administered and she underwent stereotactic endoscopic transsphenoidal excision of the pituitary adenoma within 24 h. Histopathology confirmed an anterior pituitary lesion with evidence of recent and prior haemorrhage (Fig. 2a). Pituitary hormone immunohistochemistry confirmed a prolactinoma (Fig. 2c).

## Outcome and follow-up

She reported a dramatic improvement in her vision within 24 hours. Follow-up perimetry confirmed normal visual fields. There were no adverse neurological events, cerebrospinal fluid leak or diabetes insipidus. There was no evidence of impact on the foetus.

Postoperatively, corticosteroid therapy was successfully weaned and then stopped 3 weeks later as normal morning cortisol levels were measured and she remained euthyroid.

She delivered a healthy baby boy via an uncomplicated vaginal delivery at term. She breast-fed for the first 2 weeks only.

At 14 months *post partum*, she remained well with a regular menstrual cycle. Her pituitary hormone profile was normal. Specifically, her serum prolactin level was 395 mU/l (0–536 mIU/l). Pituitary MRI demonstrated a small amount of residual pituitary tissue in the left side of the sella with no evidence of adenoma recurrence.

## Discussion

Pituitary apoplexy is an acute clinical syndrome characterised by an array of presenting features including sudden onset of headache, nausea and vomiting, meningism and sometimes altered consciousness (1) (2). Ophthalmoplegia, deterioration of visual acuity and restriction of visual fields also frequently occur (1) (2). The incidence is <10% when clinical signs and surgical or histopathological evidence are both considered; however, subclinical apoplexy is more common (1) (2). Apoplexy typically occurs in macroadenomas; however, small tumours also haemorrhage and the pathophysiology remains uncertain (1) (2) (3). There is no subtype of adenoma that has been shown to increase the risk and the event occurs in hyperfunctioning and non-functioning tumours (1). Although apoplexy occurs spontaneously in most patients (1) (2) (3), numerous risk factors have been described including hypertension, hypotension, dynamic testing of pituitary function, sudden head trauma, gonadotrophin-releasing hormone analogue therapy, dopamine agonist therapy and anticoagulation (1) (2) (3).

The clinical manifestations of pituitary apoplexy are related to the predominant underlying pathological mechanism including compression of neural structures secondary to sudden upward and lateral haemorrhagic tumour expansion, meningeal irritation and features similar to subarachnoid haemorrhage due to extravasation of blood into the subarachnoid space and hypopituitarism secondary to compression or destruction of the gland (3). The clinical appearance of pituitary apoplexy may be highly variable and the diagnosis can often be delayed or overlooked especially given that the majority of apoplectic patients have no knowledge of pre-existing pituitary disease (1) (2) (3). Partial or complete hypopituitarism is evident in most patients at presentation and prompt recognition of the diagnosis allows immediate corticosteroid replacement (1) (2) (3).

Pituitary apoplexy is rare in pregnancy with only 15 cases published in the literature (12 pituitary adenomas, two lymphocytic hypophysitis and one normal pituitary gland) (4). The pituitary gland increases in size by two- to threefold in pregnancy (5). This is a physiological adaptation mainly attributable to lactotroph hyperplasia stimulated by high oestradiol levels and usually resolves by 6 months *post partum* (6) (7).

Initial management of apoplexy includes immediate corticosteroid replacement and careful monitoring of fluid balance and electrolytes. Urgent surgery, usually by the transsphenoidal route, is indicated in patients who present with a deteriorating level of consciousness or a significant or progressive neuro-ophthalmological deficit (1) (3) (8). However, ocular paresis alone is not an indication for surgery, as this will typically improve over days to weeks (1) (8).

The management of conscious patients with absent or mild and stable neuro-ophthalmological signs is more controversial. Current guidelines are of little assistance because only level IIIB evidence exists for recommendations (8) and an improved evidence base is unlikely to be ethically possible in the future in the absence of true clinical equipoise. Historically, investigators have supported the efficacy and safety of urgent surgical intervention. They argue that early surgery performed by an experienced pituitary neurosurgeon has low morbidity and mortality and that visual acuity or field deficits can be consistently corrected or improved, especially when surgery is performed within the first week (1) (8). They also advocate that early pituitary decompression can lead to preservation of pituitary function (1) (8). However, more recent retrospective studies have found that mild neuro-ophthalmic signs tend to improve with conservative management in the majority of apoplectic patients (8) (9) (10). In addition, endocrine outcomes were similar in patients managed conservatively or by early surgical intervention (9) (10). It is these studies that question as to whether the surgical risk of cerebrospinal fluid leak, permanent diabetes insipidus and removal of normal anterior pituitary in patients with mild and stable neuro-ophthalmic signs is justified (8). Nevertheless, careful monitoring of conservatively managed patients, including daily formal assessment of visual fields and acuity, is paramount with reconsideration of surgery if there is no clear trend of improvement (8).

Similarly, there are no clear guidelines on the management of pituitary apoplexy in pregnancy. In the setting of a prolactinoma and non-apoplectic symptomatic tumour growth, most recommend re-initiation of a dopamine agonist as first-line therapy as this is generally considered to pose less risk to the mother and foetus than surgical intervention (6) (7).

Pituitary apoplexy has an unpredictable clinical course and determining which clinical situations warrant early surgery will need to take into consideration the presence and severity of neurological signs and their stability. In the absence of more evidence-based data, guidelines can only defer to clinical judgement in determining a conservative vs surgical approach in patients with mild and stable clinical signs. Clinical decision-making in patients with apoplexy, because of its infrequent nature and potentially devastating consequences, needs to be made by a senior multidisciplinary management team. Our case demonstrates that a multidisciplinary approach involving endocrinologists, obstetricians and neurosurgeons can minimise morbidity and mortality in the pregnant apoplectic patient and that early decompressive surgery is safe in the second trimester and can preserve pituitary function.

## Patient consent

Written informed consent was obtained from the patient.

## Author contribution statement

A R Hayes was responsible for drafting the manuscript. A J O'Sullivan and M A Davies were involved in critical revision of the manuscript.
